# Therapy-related MDS dissected based on primary disease and treatment—a nationwide perspective

**DOI:** 10.1038/s41375-023-01864-6

**Published:** 2023-03-17

**Authors:** Daniel Moreno Berggren, Hege Garelius, Petter Willner Hjelm, Lars Nilsson, Bengt Rasmussen, Caroline E. Weibull, Mats Lambe, Sören Lehmann, Eva Hellström-Lindberg, Martin Jädersten, Elisabeth Ejerblad

**Affiliations:** 1 Department of Medical Science, Section of Hematology, Uppsala University, Uppsala, Sweden; 2 Section of Hematology and Coagulation, Department of Specialist Medicine, Sahlgrenska University Hospital, Gothenburg, Sweden; 3 Department of Hematology, Linköping University Hospital, Linköping, Sweden; 4 Department of Hematology, Oncology and Radiation Physics, Skåne University Hospital, Lund, Sweden; 5 Department of Medicine, Faculty of Medicine and Health, Örebro University, Örebro, Sweden; 6 Clinical Epidemiology Division, Department of Medicine Solna, Karolinska Institutet, Stockholm, Sweden; 7 Department of Medical Epidemiology and Biostatistics, Karolinska Institutet, Stockholm and Regional Cancer Center Central Sweden, Uppsala, Sweden; 8 Center for Hematology and Regenerative Medicine, Department of Medicine Huddinge, Karolinska Institutet and Department of Hematology, Karolinska University Hospital, Stockholm, Sweden

**Keywords:** Myelodysplastic syndrome, Epidemiology, Prognosis

## Abstract

In this population-based study, we aimed to characterize and compare subgroups of therapy-related Myelodysplastic syndromes (t-MDS) and define the implications of type of previous treatment and primary disease. We combined data from MDS patients, diagnosed between 2009 and 2017 (*n* = 2705), in the nationwide Swedish MDS register, with several health registers. Furthermore, using matched population controls, we investigated the prevalence of antecedent malignancies in MDS patients in comparison with the general population. This first ever nationwide study on t-MDS confirms a shorter median survival for t-MDS compared to de novo MDS (15.8 months vs 31.1 months, *p* < 0.001). T-MDS patients previously treated with radiation only had disease characteristics with a striking resemblance to de novo-MDS, in sharp contrast to patients treated with chemotherapy who had a significantly higher risk profile. IPSS-R and the WHO classification differentiated t-MDS into different risk groups. As compared with controls, MDS patients had a six-fold increased prevalence of a previous hematological malignancy but only a 34% increased prevalence of a previous solid tumor. T-MDS patients with a previous hematological malignancy had a dismal prognosis, due both to mortality related to their primary disease and to high-risk MDS.

## Introduction

Myelodysplastic syndromes (MDS) diagnosed after exposure to chemotherapy and/or radiation are classified as therapy-related (t)-MDS and constitute 10–20% of all MDS [1]. Together with t-acute myeloid leukemia (AML) and t-myelodysplastic/myeloproliferative neoplasms (MDS/MPN), t-MDS is included in the entity therapy-related myeloid neoplasms (t-MN) in the 2016 World Health Organization (WHO) classification [2]. Compared to de novo-MDS, t-MDS patients have higher-risk clinical characteristics, including more cytogenetic aberrations, high-risk mutations, as well as shorter survival [1, 3, 4]. A growing numbers of cancer survivors, longer life-expectancy, and the increased use of adjuvant chemotherapy are expected to increase the future number of t-MN patients [5, 6].

The understanding of the pathogenesis of t-MN has evolved substantially during the last decade. Historically regarded as a direct consequence of cytotoxic treatment leading to DNA damage in hematopoietic stem cells, the field have shifted to a more complex understanding of the etiology of t-MN. Clonal selection of pre-existing mutant clones [7–9], inherited cancer predisposition [10], and the disruption of the normal bone marrow microenvironment all contribute to the development of t-MN [11].

In this study, based on the Swedish MDS register (SMDSR) and several national health registers we characterize and compare subgroups of t-MDS. Furthermore, we investigate the prevalence of malignancies preceding MDS and compare this with the general population. With our nationwide population-based data, we aim to contribute to the efforts in redefining t-MDS and its prognostic implications.

## Materials and methods

### Patients and data sources

All patients with a MDS diagnosis registered in the SMDSR 2009-2017 were included. The register includes detailed clinical data as described in detail elsewhere [12]. Mutational data was not available. All Swedish hospitals diagnosing MDS (*n* = 67) report to the register, see Supplementary Fig. 1 for a list of contributing sites and their geographical distribution. During the period under study, the completeness of the SMDSR was 97% compared to the Cancer Register to which reporting is mandatory [13]. T-MDS was defined according to the WHO 2016 classification as patients who had received any type of chemotherapy and/or radiation prior to the diagnosis of MDS.

For the purpose of the present study, we generated a dataset based on individual level record linkage between several registers with national coverage, including SMDSR, the National Patient Register, the Cancer Register, and the Cause of Death Register. These registers are described in detail in the appendix (supplemental material). The primary disease was defined as a disease diagnosed at any time before MDS diagnosis for which chemotherapy and/or radiation was given. In cases where there were multiple possible primary diseases, the most likely one was selected, based on data from all applicable registers, treatment of the particular disease and the time span between the primary disease and the diagnosis of MDS. Data on type or doses of chemotherapy and radiation were not available.

Comorbidity was estimated with Charlson comorbidity index (CCI) [14] using a recently published coding algorithm [15], including diagnoses 10 years preceding MDS diagnosis. To be able to compare comorbidity in de novo-MDS and t-MDS patients, we excluded malignancies. To compare the prevalence of malignant diseases between MDS patients and the general population, controls free from MDS and MDS/MPN at time of case diagnosis were randomly selected from the National Population Register and matched 1:5 on sex, age, and county of residence.

Causes of death were obtained up to December 31, 2018. Patients were followed for death or emigration until November 20, 2019. This study was approved by the ethics committee of Uppsala University (2014/176).

### Statistical analysis

To assess the distribution of baseline patient characteristics, standard descriptive techniques were used, including chi-squared test and Wilcoxon rank sum test. Overall survival (OS) was estimated by the Kaplan–Meier method and compared using the log-rank test. Relative mortality was analyzed with Cox proportional hazards models, yielding hazard ratios (HRs) with 95% confidence intervals (CIs). Unadjusted logistic regression models were fitted to compare the likelihood of previous malignant disease between MDS patients and their matched controls, yielding odds ratios (ORs) and 95% CIs. A *p*-value of less than 0.05 was considered statistically significant. All analyses were performed using Stata 16 (StataCorp, TX, USA) and SPSS Statistics for Windows, Version 28 (IBM, NY, USA).

## Results

### Study population and patient characteristics

A total of 2705 patients were included in this study, 423 (16%) of whom were classified as t-MDS. The median follow-up for all patients was 26 months (range 0–130) and for surviving patients 56 months (range 23–130). Our dataset also included 13,509 matched controls.

Table 1 and Supplementary Table 1 show characteristics for patients with de novo-MDS, t-MDS and subgroups of t-MDS. A higher proportion of t-MDS patients were found in the high (24%) and very high (26%) Revised International Prognostic Scoring System (IPSS-R) groups compared to de novo-MDS (15% and 14%, respectively) (*p* < 0.001), a major contributing factor was the large number of t-MDS patients with high-risk cytogenetics (39% with poor or very poor cytogenetic risk group). The comorbidity burden was similar in patients with t-MDS and de novo-MDS, with a mean CCI of 0.84 and 0.72, respectively (*p* = 0.55) (data not shown).

**Table 1:** Patient and disease characteristics among patients with de novo and t-MDS and within subgroups of t-MDS by to the type of previous cytotoxic treatment.

	De novo-MDS *n* = 2283 (84%)	t-MDS *n* = 423 (16%)	Subgroups of t-MDS		
			Chemotherapy *n* = 239 (56%)	Radiation *n* = 105 (25%)	Chemotherapy and Radiation *n* = 79 (19%)
Median survival, months (95% CI)	31.1 (29.0–33.3)	15.8 (13.6–18.1)	13.3 (10.6–16.1)	34.8 (24.6–45.1)	9.0 (3.6–14.4)
Sex					
Female	922 (40%)	194 (46%)	106 (44%)	49 (47%)	39 (49%)
Male	1360 (60%)	229 (54%)	133 (56%)	56 (53%)	40 (51%)
Age at diagnosis, years					
<60	216 (9%)	33 (8%)	20 (8%)	1 (1%)	12 (15%)
60–74	815 (36%)	200 (47%)	123 (51%)	37 (35%)	40 (51%)
≥75	1251 (55%)	190 (45%)	96 (40%)	67 (64%)	27 (34%)
Median age at diagnosis, years (range)	76 (16–97)	73 (18–92)	72 (18-91)	77 (56-92)	72 (39-89)
WHO subgroup					
MDS-SLD	185 (8%)	23 (5%)	13 (5%)	8 (8%)	2 (3%)
MDS-MLD	718 (32%)	121 (29%)	61 (26%)	36 (34%)	24 (30%)
MDS-RS*	273 (12%)	21 (5%)	8 (3%)	13 (12%)	0 (0%)
MDS-EB-1	350 (15%)	82 (19%)	46 (19%)	20 (19%)	16 (20%)
MDS-EB-2	397 (17%)	97 (23%)	64 (27%)	11 (11%)	22 (28%)
MDS with isolated del(5q)	93 (4%)	15 (4%)	11 (5%)	2 (2%)	2 (3%)
MDS-U	266 12%)	64 (15%)	36 (15%)	15 (14%)	13 (16%)
Medullary blast count, %					
<2	730 (33%)	108 (27%)	60 (26%)	35 (35%)	13 (17%)
2–4.9	667 (31%)	110 (27%)	57 (25%)	29 (29%)	24 (31%)
5–9.9	384 (18%)	91 (22%)	50 (22%)	22 (22%)	19 (24%)
≥10	407 (19%)	98 (24%)	61 (27%)	15 (15%)	22 (28%)
Missing	94	16	11	4	1
IPSS-R cytogenetic risk group					
Very good	165 (9%)	25 (8%)	14 (8%)	8 (9%)	3 (5%)
Good	995 (56%)	125 (38%)	61 (33%)	48 (57%)	16 (26%)
Intermediate	267 (15%)	51 (16%)	29 (16%)	14 (17%)	8 (13%)
Poor	122 (7%)	44 (13%)	30 (16%)	5 (6%)	9 (15%)
Very poor	216 (12%)	84 (26%)	49 (27%)	10 (12%)	25 (41%)
Missing	517	94	56	20	18
IPSS-R					
Very low	335 (19%)	40 (12%)	15 (9%)	21 (24%)	4 (7%)
Low	563 (32%)	72 (22%)	41 (23%)	24 (28%)	7 (12%)
Intermediate	337 (19%)	52 (16%)	30 (17%)	17 (20%)	5 (8%)
High	253 (15%)	77 (24%)	43 (24%)	14 (16%)	20 (33%)
Very high	250 (14%)	83 (26%)	48 (27%)	11 (13%)	24 (40%)
Missing	544	99	62	18	19
Red blood cell transfusion dependency at diagnosis					
Yes	1033 (45%)	232 (55%)	138 (58%)	44 (42%)	50 (63%)
No	1242 (55%)	190 (45%)	100 (42%)	61 (58%)	29 (37%)
Missing	7	1	1	0	0
Platelet transfusion dependency at diagnosis					
Yes	125 (6%)	47 (11%)	29 (12%)	5 (5%)	13 (16%)
No	2142 (94%)	375 (89%)	209 (88%)	100 (95%)	66 (84%)
Missing	15	1	1	0	0
CCI					
0	1360 (60%)	222 (53%)	117 (49%)	57 (54%)	48 (61%)
1	506 (22%)	108 (26%)	58 (24%)	30 (29%)	20 (25%)
2	230 (10%)	51 (12%)	35 (15%)	12 (11%)	4 (5%)
>2	186 (8%)	42 (10%)	29 (12%)	6 (6%)	7 (9%)

*CI* Confidence interval, *MDS-SLD* MDS with single lineage dysplasia, *MDS-MLD* MDS with multilineage dysplasia, *MDS-RS* MDS with ring sideroblasts, *MDS-EB* MDS with excess blasts, *MDS-U* MDS unclassifiable, *IPSS-R* International Prognostic Scoring System Revised, *CCI* Charlson Comorbidity Index.

*Including both MDS-RS SLD and MDS-RS-MLD.

In patients with t-MDS, 56% had been treated with chemotherapy only for their primary disease, 25% with radiation only and 19% with both (Table 1). De novo-MDS and t-MDS patients treated with radiation only had similar distribution of transfusion dependency, blast count, and cytogenetic risk group (*p* > 0.05 for all three variables). By contrast, t-MDS treated with chemotherapy only or chemotherapy in combination with radiation had transfusion dependency, blast count, and cytogenetic risk group with a significantly higher risk profile than de novo-MDS (*p* < 0.05 for all three variables). A higher proportion of t-MDS patients with a hematological malignancy as their primary disease had IPSS-R high risk or very high risk (67%) compared to solid tumor as primary disease (37%) or non-malignant primary disease (40%) (*p* < 0.001). Particularly, adverse cytogenetics contributed to the high scores according to IPSS-R for patients with a previous hematological disease (Supplementary Table 1).

The primary disease was a solid tumor in 176 patients (42%), among whom 95 (54%) had received radiation only (Table 2). A hematological malignancy was the primary disease in 160 patients (38%) most of these patients had received chemotherapy only (78%). A non-malignant disease was the primary disease in 63 patients (15%). Of the patient with a non-malignant disease 46 (78%) had received a prescription of methotrexate (data not shown). Information regarding primary disease was unavailable for 24 patients (6%). The median latency time between the diagnosis of the primary disease and MDS was 6.6 years and 6.5 years for solid tumors and hematological malignancy, respectively.

**Table 2:** Types of primary diseases in t-MDS, treatment for the primary disease, median latency and OS.

Primary disease	*n*	Chemotherapy	Radiation	Chemotherapy and radiation	Median latency time, years (min-max)	Median OS, months (95% CI)
Solid tumors*	176	37 (21%)	95 (54%)	45 (25%)	6.6 (0.4–48.9)	22 (15.9–28.1)
Prostate	50	3 (6%)	44 (88%)	3 (6%)	7.9 (0.9–19.8)	41 (25.3–56.7)
Breast	40	7 (18%)	19 (48%)	14 (35%)	7.7 (0.8–21.7)	41 (24.4–58.4)
Uterine	20	1 (5%)	9 (45%)	10 (50%)	8.1 (0.8–21.9)	12 (0–27.3)
Lung	15	6 (40%)	5 (33%)	4 (27%)	3.9 (0.4–8.6)	14 (9.4–18.6)
Colon/rectal	14	7 (50%)	4 (29%)	3 (21%)	8.0 (0.4–13.4)	57 (0–120.5)
Head and neck	6	0	6 (100%)	0	3.8 (0.4–7.5)	29 (0–59.0)
Ovarian	6	5 (83%)	0	1 (17%)	3.0 (0.4–13.0)	18 (2.4–33.6)
CNS and eye	7	0	2 (29%)	5 (71%)	4.8 (1.9–15.3)	10 (4.9–15.1)
Hematological malignancies	160	124 (78%)	2 (1%)	34 (21%)	6.5 (0.3–32.0)	9 (6.9–11.1)
NHL**	71	53 (75%)	2 (3%)	16 (23%)	5.7 (0.3–25.6)	10 (7.0–13.0)
Myeloma	19	12 (63%)	0	7 (37%)	5.3 (1.7–21.6)	6 (2.8–9.2)
AML	18	17 (94%)	0	1 (6%)	5.0 (1.1–18.8)	15.0 (4.6–25.4)
ET	14	13 (93%)	0	1 (7%)	7.4 (1.3–22.0)	7 (0–27.2)
CLL	11	10 (91%)	0	1 (9%)	9.6 (1.4–15.6)	4 (1.4–6.6)
Hodgkin lymphoma	9	2 (22%)	0	7 (78%)	14.8 (1.8–32.0)	14 (0–37.4)
Polycythemia Vera	9	8 (89%)	0	1 (11%)	5.9 (2.2–14.8)	7 (1.2–12.8)
Myelofibrosis/MPN NOS	6	6 (100%)	0	0	8.0 (3.3–14.0)	4 (0–12.4)
ALL	3	3 (100%)	0	0	2.8 (1.5-18.0)	49.0 (0–111.4)
Non-malignant diseases***	63	62 (99%)	1 (1%)	0	–	26 (7.8–44.2)
Rheumatoid arthritis	30	30 (100%)	0	0	–	33.0 (8.6–57.4)
Psoriasis/psoriatic arthritis	12	12 (100%)	0	0	–	18.0 (9.5–26.5)
SLE/systemic inflammatory disease/vasculitis	10	10 (100%)	0	0	–	13 (0–36.2)
IBD	5	5 (100%)	0	0	–	16 (3.1–28.9)
Other	5	4 (80%)	1 (20%)	0	–	44 (14.1–73.9)
Unknown non-malignant	1	1 (100%)	0	0	–	–
Unknown	24	16 (67%)	7 (30%)	1 (4%)	–	26.0 (1.7–50.3)

*OS* Overall survival, *CNS* Central nervous system, *NHL* Non-Hodgkin lymphoma, *AML* acute myeloid leukemia, *ET* essential thrombocythemia, *CLL* chronic lymphocytic leukemia, *MPN NOS* Myeloproliferative neoplasia not otherwise specified, *ALL* acute lymphocytic leukemia, *SLE* systemic lupus erythematosus, *IBD* Inflammatory bowel disease.

*Not shown 3 cases of bladder cancer, testicular cancer and cervical cancer, 2 cases of anal cancer, cancer of the small intestine and sarcoma, 1 case of esophageal, skin and kidney cancer.

**Including 4 cases of Waldenstrom’s disease.

***Not shown one case of bullous pemphigoid, nephritis, autoimmune hemolytic anemia, polymyalgia rheumatica, and thyrotoxicosis (treated with radioiodine).

### Overall survival

**Fig. 1:**
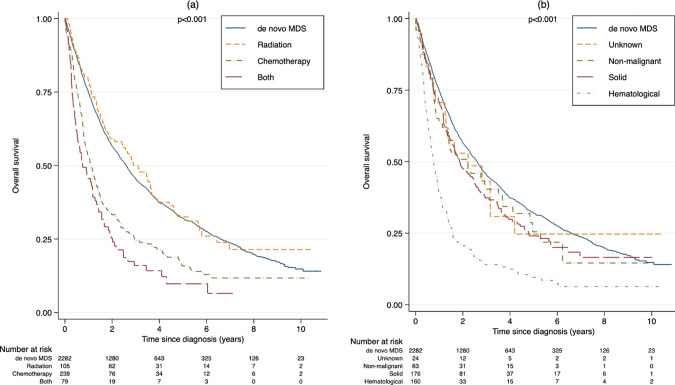
OS of de novo-MDS and subgroups of t-MDS. **a** OS by different treatments for the primary disease. **b** OS by type of primary disease.

The median OS of t-MDS was 15.8 months, compared to 31.1 months for de novo-MDS (*p* < 0.001) (Table 1). Patients treated with chemotherapy or chemotherapy and radiation in combination had significantly shorter survival (13.3 and 9.0 months, respectively) than patients treated with radiation only (34.8 months) (*p* < 0.001) (Table 1, Fig. 1a).

T-MDS patients with a previous non-malignant disease and a previous solid tumor had longer OS, (26.1 and 22.3 months) compared with patients with a history of a hematological malignancy (9.0 months) (*p* < 0.001) (Fig. 1b, Supplementary Table 1.).

### Survival according to IPSS-R and WHO classification

**Fig. 2:**
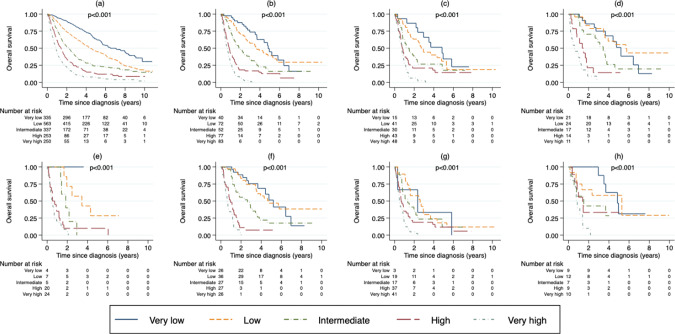
OS by IPSS-R risk group. **a** OS of patients with de novo-MDS. **b** OS of all patients with t-MDS. **c** OS of patients with t-MDS treated with chemotherapy only. **d** OS of patients with t-MDS treated with radiation only. **e** OS of patients with t-MDS treated with chemotherapy in combination with radiation. **f** OS of patients with t-MDS with a solid tumor as a primary disease. **g** OS of patients with t-MDS with a hematological malignancy as a primary disease. **h** OS of patients with t-MDS with a non-malignant disease as a primary disease.

IPSS-R effectively discriminated different risk groups in both de novo-MDS and t-MDS (Fig. 2a, b) and in subgroup analyses of patients treated with chemotherapy only, radiation only, or a combination of chemotherapy and radiation (Fig. 2c–e). Furthermore, IPSS-R could separate risk groups for patients with solid tumor and non-malignant disease as the primary disease, but to a lesser extent for hematological disease (Fig. 2f–h). In Supplementary Fig. 2, we show that the survival in each IPSS-R risk group was similar for de novo and t-MDS in the very low, low and intermediate risk group but shorter for t-MDS in the high and very high risk group.

**Fig. 3:**
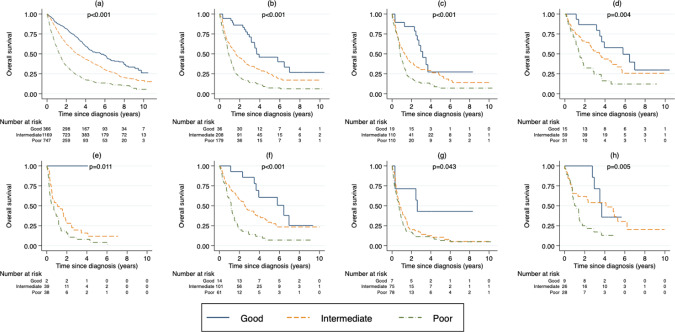
OS by WHO risk group. **a** OS of patients with de novo-MDS. **b** OS of all patients with t-MDS. **c** OS of patients with t-MDS treated with chemotherapy only. **d** OS of patients with t-MDS treated with radiation only. **e** OS of patients with t-MDS treated with chemotherapy in combination with radiation. **f** OS of patients with t-MDS with a solid tumor as a primary disease. **g** OS of patients with t-MDS with a hematological malignancy as a primary disease. **h** OS of patients with t-MDS with a non-malignant disease as a primary disease.

The OS for de novo-MDS and t-MDS according to WHO classification is illustrated in Fig. 3a, b. Since there were a limited number of patients in each WHO group, these were combined according to their median survival. MDS with isolated del(5q) and MDS-RS were combined into a good-risk group, MDS-SLD, MDS-MLD, and MDS-U were combined into an intermediate group and MDS-EB1 and MDS-EB2 into a poor-risk group. There was a difference in survival in t-MDS according to the WHO-based risk groups, good vs intermediate (*p* < 0.002) and intermediate vs poor (*p* < 0.001). The WHO classification could also discriminate different risk groups in subgroups based on type of cytotoxic treatment (Fig. 3c–e). In subgroups based on type of primary disease, the WHO classification was able to separate different risk groups in patients with solid tumors, but to a lesser extent for patients with hematological malignancies (Fig. 3f–h). The survival within the WHO-based risk groups was shorter for t-MDS compared to de novo MDS in the intermediate and poor risk group (Supplementary Fig. 3).

### Uni- and multivariable analysis of all-cause mortality

**Fig. 4:**
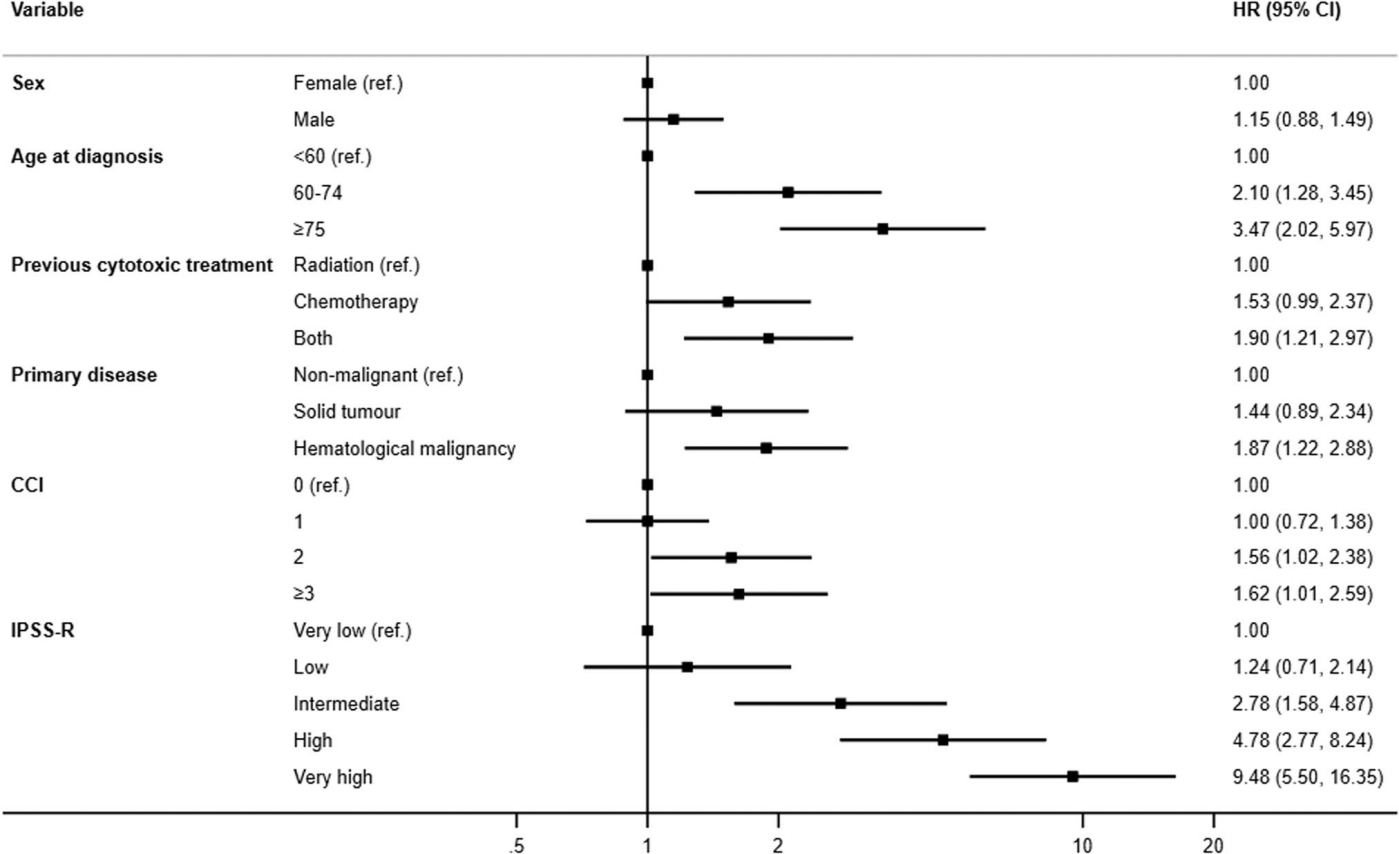
Multivariate analyses of the all-cause mortality of t-MDS patients by patient and disease characteristics.

In univariable analysis of the group with t-MDS, type of previous cytotoxic treatment, type of primary disease, WHO risk group, comorbidity as measured with CCI, medullary blast count, cytogenetic risk group, risk group according to IPSS-R, red blood cell and platelet-transfusion dependency were all associated with survival (Table 3). In multivariable analysis, age group, type of previous cytotoxic treatment, type of primary disease, CCI, and risk group according to IPSS-R were independently associated with survival (Fig. 4).

**Table 3:** Univariate analysis of the all-cause mortality of t-MDS patients by patient and disease characteristics.

	Median OS, 95% CI, months	HR, 95% CI	p*
Total	15.8 (13.6–18.1)		
Sex			
Female	17.9 (14.0–21.8)	1	0.288
Male	13.7 (11.1–16.4)	1.12 (0.91–1.39)	
Age at diagnosis, years			
<60	14.1 (10.6–17.6)	1	0.146
60–74	17.9 (15.1–20.7)	1.29 (0.82–2.03)	
≥75	13.4 (9.5–17.4)	1.49 (0.95–2.36)	
Previous cytotoxic treatment			
Radiation	34.8 (24.6–45.1)	1	<0.001
Chemotherapy	13.3 (10.6–16.1)	1.78 (1.36–2.33)	
Both	9.0 (3.6–14.4)	2.35 (1.69–3.26)	
Primary disease			
Non-malignant	26.1 (8.7–43.5)	1	<0.001
Solid tumor	22.3 (16.2–28.3)	1.03 (0.73–1.44)	
Hematological malignancy	9.0 (7.1–10.9)	2.04 (1.46–2.86)	
WHO subgroup			
MDS with isolated del(5q)	44.4 (39.2–49.6)	1	<0.001
MDS-RS**	47.2 (11.1–83.4)	1.55 (0.59–4.03)	
MDS-SLD	28.1 (10.8–45.3)	2.39 (0.93–6.10)	
MDS-MLD	19.6 (10.9–28.3)	2.57 (1.12–5.88)	
MDS-U	16.7 (5.4–28.0)	3.31 (1.42–7.71)	
MDS-EB-1	11.9 (8.3–15.5)	4.12 (1.79–9.51)	
MDS-EB-2	9.5 (7.9–11.1)	5.70 (2.49–13.05)	
Medullary blast count, %			
<2	32.5 (26.0–39.0)	1	<0.001
2–4.9	19.9 (14.1–25.7)	1.25 (0.91–1.70)	
5–9.9	11.0 (7.6–14.5)	1.88 (1.37–2.59)	
≥10	9.2 (7.5–10.9)	2.59 (1.90–3.52)	
IPSS-R cytogenetic risk group			
Very good	57.3 (35.4–79.2)	1	<0.001
Good	37.3 (25.7–49.0)	1.50 (0.82–2.74)	
Intermediate	16.0 (7.7–24.3)	2.55 (1.34–4.85)	
Poor	11.6 (6.2–16.9)	3.55 (1.84–6.84)	
Very poor	7.6 (5.9–9.5)	9.68 (5.18–18.11)	
IPSS-R			
Very low	58.2 (49.3–67.0)	1	<0.001
Low	44.0 (32.4–55.6)	1.27 (0.76–2.14)	
Intermediate	22.1 (11.9–32.4)	2.29 (1.35–3.88)	
High	10.3 (8.2–12.3)	4.47 (2.72–7.33)	
Very high	7.7 (5.4–9.9)	9.47 (5.73–15.67)	
RBC transfusion dependency at diagnosis			
No	33.2 (22.5–43.9)	1	<0.001
Yes	9.14 (7.7–10.6)	2.48 (1.99–3.11)	
Platelet transfusion dependency at diagnosis			
No	17.2 (14.7–19.7)	1	<0.001
Yes	8.4 (5.2–11.6)	2.24 (1.62–3.09)	
CCI			
0	16.7 (12.7–20.7)	1	0.009
1	18.2 (13.4–23.0)	0.98 (0.75–1.27)	
2	9.9 (6.2–13.5)	1.47 (1.05–2.04)	
>2	9.6 (3.3–15.9)	1.59 (1.11–2.27	

*OS* Overall survival, *HR* hazard ratio, *CI* Confidence interval, *MDS-SLD* MDS with single lineage dysplasia, *MDS-MLD* MDS with multilineage dysplasia, *MDS-RS* MDS with ring sideroblasts, *MDS-EB* MDS with excess blasts, *MDS-U* MDS unclassifiable, Charlson Comorbidity Index, *IPSS-R* International Prognostic Scoring System Revised, *RBC* Red blood cell.

*From a Wald test.

**Including both MDS-RS SLD and MDS-RS-MLD.

### Causes of death

Patients with a previous hematological malignancy had their primary malignancy more often stated as their cause of death (45%) than patients with a previous solid tumor (15%) (Supplementary Table 2). Patients with previous Hodgkin’s lymphoma, chronic lymphocytic leukemia, and myeloma had their primary disease stated as underlying cause of death in their death certificate in more than 50% of cases.

### Prior history of cancer in cases and controls

We observed in total 737 malignancies in 565 (20.9%) MDS patients and 2366 malignancies in 2217 (16.4%) controls. The prevalence of prior malignancies, latency between the previous malignancies and MDS and ORs are presented in Table 4. MDS patients were more likely to have had a solid tumor than controls (OR = 1.34, 95% CI: 1.21–1.49). The highest ORs for solid tumors were found for penile and testicular cancer (OR = 2.39, 95% CI: 1.12–5.08), lung, mediastinal, pleural and myocardial cancers (OR 1.92 95% CI 1.19–3.11) and head and neck cancer (OR = 1.88, 95% CI: 1.09–3.23). There was a six-fold increase for antecedent hematological malignancies in MDS patients (OR = 6.09, 95% CI: 4.87–7.61). All types of hematological malignancies were overrepresented, the most common were non-Hodgkin lymphoma, multiple myeloma and essential thrombocythemia (ET). The highest ORs for hematological malignancy were found for AML (OR = 28.5, 95% CI: 8.34–97.2), Myelofibrosis/MPN NOS (OR = 22.5, 95% CI: 4.87–104.4) and ET (OR = 18.1, 95% CI: 6.71–48.8).

**Table 4:** Prevalence and time since initial diagnosis (latency) of at least one previous malignant disease among MDS patients (both t- and de novo-MDS) and controls.

	MDS patients *N* (%)	Controls *N* (%)	OR^a^ (95% CI)	Median latency^b^ (IQR)
Total	2705 (16.7)	13,509 (83.3)	–	–
Solid tumor	565 (20.9)	2217 (16.4)	1.34 (1.21–1.49)	–
Prostate (among males)	161 (10.1)	736 (9.3)	1.10 (0.92–1.32)	6.9 (3.5–10.4)
Breast cancer	88 (3.3)	319 (2.4)	1.39 (1.09–1.77)	11.2 (6.2–20.1)
Intestinal cancer	77 (2.9)	314 (2.3)	1.23 (0.96–1.59)	–
Colon	56 (2.1)	167 (1.2)	1.69 (1.24–2.29)	6.2 (2.6–13.6)
Rectal and anal	15 (0.6)	106 (0.8)	0.71 (0.41–1.21)	8.1 (3.9–13.9)
Other intestinal	7 (0.3)	45 (0.3)	0.78 (0.35–1.72)	8.6 (5.0–22.5)
Non-melanoma skin	63 (2.3)	222 (1.6)	1.43 (1.08–1.89)	3.6 (1.6–6.6)
Gynecological* (among females)	50 (4.5)	152 (2.7)	1.67 (1.21–2.32)	–
Uterine	35 (3.1)	98 (1.8)	1.81 (1.22–2.68)	9.1 (3.4–12.4)
Ovarian	6 (0.5)	24 (0.4)	1.25 (0.51–3.07)	3.0 (1.5–21.4)
Cervical	8 (0.7)	23 (0.4)	1.74 (0.78–3.91)	26.2 (16.3–38.6)
Urinary tract	37 (1.4)	181 (1.3)	1.02 (0.72–1.46)	9.2 (3.8–15.9)
Melanoma	34 (1.3)	161 (1.2)	1.06 (0.73–1.53)	14.4 (6.0–25.6)
Other malignancies**	26 (1.0)	68 (0.5)	1.92 (1.22–3.02)	4.1 (2.3–6.2)
Lung, mediastinal, pleural, myocardium	23 (0.9)	60 (0.4)	1.92 (1.19–3.11)	3.8 (1.2–4.8)
Central nervous system and eye	19 (0.7)	79 (0.6)	1.20 (0.73–1.99)	12.2 (4.8–17.6)
Head and neck	18 (0.7)	48 (0.4)	1.88 (1.09–3.23)	12.7 (5.8–19.0)
Kidney and adrenal	17 (0.6)	53 (0.4)	1.61 (0.93–2.78)	4.7 (2.0–18.6)
Parathyroid and thyroid	12 (0.4)	59 (0.4)	1.02 (0.55–1.89)	15.6 (7.4–30.2)
Penile and testicular (among males)	10 (0.6)	21 (0.3)	2.39 (1.12–5.08)	10.3 (5.2–35.4)
Hematological malignancy	172 (6.4)	149 (1.1)	6.09 (4.87–7.61)	–
Non-Hodgkin lymphoma***	74 (2.7)	75 (0.6)	5.04 (3.64–6.96)	6.5 (3.0–11.5)
Multiple myeloma	19 (0.7)	19 (0.1)	5.02 (2.66–9.50)	5.3 (3.4–10.4)
Essential thrombocythemia	18 (0.7)	5 (0.0)	18.1 (6.71–48.8)	7.4 (3.6–15.0)
Acute myeloid leukemia	17 (0.6)	3 (0.0)	28.5 (8.34–97.2)	5.5 (3.8–7.8)
Chronic lymphocytic leukemia	12 (0.4)	28 (0.2)	2.15 (1.09–4.22)	6.8 (4.4–11.1)
Hodgkin lymphoma	10 (0.4)	6 (0.0)	8.35 (3.03–23.0)	17.2 (7.1–29.5)
Polycythemia Vera	10 (0.4)	10 (0.1)	5.01 (2.08–12.0)	6.5 (4.6–17.5)
Myelofibrosis/MPN NOS	9 (0.3)	2 (0.0)	22.5 (4.87–104.4)	5.1 (3.6–8.3)
Acute lymphocytic leukemia	3 (0.1)	1 (0.0)	15.0 (1.56–144.2)	–
Chronic myeloid leukemia	2 (0.1)	1 (0.0)	9.99 (0.91–110.3)	–

Odds ratios (ORs) and 95% confidence intervals (CIs) as measures of likelihood of prevalent cancer between patients and controls (=ref).

*IQR* Inter-quartile range, *OR* Odds ratio, *CI* Confidence interval, *MPN NOS* Myeloproliferative neoplasm not otherwise specified.

*Including, besides those specified, fallopian tube, vaginal, vulvar, and gynecological cancer NOS.

**Including sarcoma and osteosarcoma, hepatic, pancreatic, gallbladder and bile duct cancer.

***Including Waldenstrom’s disease.

^a^Estimated from unadjusted logistic regression models.

^b^Among MDS patients only.

## Discussion

In this large nationwide population-based study on 2705 patients with MDS, including 423 patients with t-MDS, we were able to examine clinical characteristics in detail and found significant differences between subgroups of t-MDS. One of our most important findings is that t-MDS patients with previous cytotoxic treatment in the form of radiation only, have clinical characteristics and prognosis comparable to de novo-MDS. They have transfusion dependency, blast count and a cytogenetic risk profile with a striking resemblance to de novo-MDS, in sharp contrast to patients treated with chemotherapy or a combination of chemotherapy and radiation.

Previous studies, including patients from earlier time periods, indicated that patients treated with radiation only that developed MDS had a high-risk disease and short survival [3, 16, 17]. Corroborating results from other more recent studies, we found that prior cytotoxic treatment with radiation only is associated with a prognosis similar to de novo-MDS [18, 19]. One explanation for this might be that the field of radiation therapy has moved to more conformal techniques leading to a decrease in the exposure to the bone marrow, this is particularly true in lymphoma treatments and in radiation to the pelvis [20, 21].

Early studies found a very high percentage of high-risk features and a uniform poor prognosis among t-MDS patients, reporting that survival was not affected by WHO subgroups or blast percentages [3, 22]. However, as in several other more recent studies, our data shows a more heterogeneous result with a fairly large group having a normal karyotype and being low risk according to IPSS-R [1, 16, 23]. Prognostic factors such as blast percentage, transfusion dependency and particularly cytogenetic risk group, were highly predictive of OS in our cohort of t-MDS. In unadjusted analysis, age was not associated with survival but in adjusted analysis it was. We believe that the reason for this is that high risk variables such as chemotherapy and previous hematological malignancy was more common among younger patients and low risk variables such as radiation was more common in older patients. Our group and others have previously reported that IPSS-R is a powerful prognostic tool for t-MDS [1, 12, 23]. Our present study shows that this also is true for all subgroups based on type of cytotoxic treatment and type of primary disease with the exception of patients with previous hematological malignancies.

In our study, the WHO classification discriminated different risk groups in all subgroups except in patients with a previous hematological malignancy. Kuendgen et al have recently in a large collaborative study on t-MDS showed that t-MDS is as heterogeneous as de novo MDS, moreover, they showed that IPSS-R and the WHO classification effectively risk classifies t-MDS and our results are in line with this [23]. We agree in their conclusion that t-MDS should be risk stratified with available prognostic tools.

The new prognostic scoring system, IPSS-M has recently been published [24]. It incorporates mutational data with the classical IPSS-R parameters and the cohort from which it was developed included around 8% of t-MDS patients [24]. When outcomes in each risk group of IPSS-M were compared between de novo and t-MDS they were similar. In our analysis of each risk group of IPSS-R the survival was shorter for t-MDS in the higher risk groups. This indicates that IPSS-M better than IPSS-R accounts for the high risk features of t-MDS and highlights the importance of a molecular evaluation in t-MDS. It is of great value that t-MDS is included in the IPSS-M which enables a correct individual prognosis for t-MDS patients and improves the possibility to include t-MDS in future clinical trials.

Two suggestions for updated classifications of myeloid neoplasms have recently been published [25, 26]. In one of these, the group therapy-related myeloid neoplasm is omitted and first priority is given to classify the therapy-related disease according to morphologic and genetic features as for de novo disease [26]. Our results that classification and prognostication for de novo MDS is effective in t-MDS are in line with those recommendations. In the other suggestion, the authors specify that only exposure to large field radiation should be considered causing t-MN [25]. In our study, we did not have information on radiation fields. We can only speculate that many patients treated with radiation only had received smaller radiation fields. However, our results suggest that t-MDS patients treated with any kind of radiation only should be considered as de novo-MDS with regard to prognostication and treatment choices. Another proposed update is that methotrexate does no longer qualify as a cause of t-MN [25]. We lack information on type of given chemotherapy for the primary condition, but we had access to data from the prescription register. We can conclude that patients with t-MDS with a non-malignant disease, of whom a majority had been treated with methotrexate, had a prognosis similar to de novo-MDS. This finding supports omitting methotrexate as a cause of t-MDS.

Our results show that mortality from the primary disease is substantial, with the highest mortality observed in patients with a previous hematological malignancy. This group of patients have a dismal prognosis with only nine months median OS. Besides a high risk of dying from their primary disease, most of them have high-risk MDS. IPSS-R and WHO classification were less effective in discriminating risk groups in t-MDS with a previous hematological disease. Based on our findings we can conclude that both primary disease and type of cytotoxic treatment strongly influence survival and that these additional variables should be considered in the prognostication of t-MDS.

One part of our study was conducted with a case-control approach comparing the history of prior malignancies between MDS patients and controls. Malignancies that usually include chemotherapy in their treatment such as colon, gynecological cancers and head and neck cancers had higher ORs, while malignancies usually treated with radiation only, such as prostate cancer, rectal cancer and thyroid cancers, had a similar prevalence in MDS patients and controls. However, firm conclusions are hard to draw due to the lack of information on the type of treatment given in the controls. The very high ORs observed for hematological malignancies are striking, particularly for myeloid malignancies. There might be a few cases with AML and MPNs where diagnostic difficulties and overlaps with MDS exist, but the long latency suggests that they represent separate previous conditions. Shared pathophysiological mechanisms and risk factors such as clonal hematopoiesis exists between the MPNs, AML, and MDS and can represent an explanation in addition to the therapy for the primary disease [27, 28]. Other non-myeloid hematological malignancies such as lymphoma and myeloma were also highly overrepresented in MDS patients. These malignancies are often treated with high doses of chemotherapy, which might lead to higher risk of t-MDS than other malignancies [29–31]. Autologous hematopoietic cell transplantation is used to treat both lymphomas and myeloma and is known to be associated with the development of t-MDS [32].

Strengths of our study includes its large size, based on virtually complete nationwide data from several high-quality population-based health registers and the availability of matched population controls. Limitations included the absence of information on details of the prior malignancy, including treatment such as specific types or doses of chemotherapy, dose of radiation or radiation field.

To the best of our knowledge, this is the first nationwide epidemiological study on t-MDS including analyses of different subgroups based on primary disease and type of therapy. Our findings show that primary disease and type of cytotoxic treatment strongly influence clinical characteristics and prognosis. T-MDS patients with previous cytotoxic treatment in the form of radiation only have clinical characteristics and prognosis comparable to de novo-MDS and should be viewed as de novo-MDS with regard to prognostication and treatment. We conclude that genetic and morphologic classification as well as risk stratification intended for de novo-MDS is meaningful in t-MDS and we suggest that t-MDS should be classified the same way de novo MDS is but with recognition that type of prior disease and cytotoxic treatment affects the prognosis. This is of particular importance in the group with a previous hematological malignancy where the outcomes are dismal. Taken together, our findings provide further evidence of the importance of an individualized approach in the management of t-MDS.

## Supplementary information

Supplementary Material

## Data Availability

The datasets generated and/or analysed in the current study are not publicly available due to privacy concerns and limitations from the ethical review board but may be available from the corresponding author upon request.
