# Supplementary material for: Therapy-related MDS dissected based on primary disease and treatment—a nationwide perspective
Source: Leukemia. 2023 Mar 17;37(5):1103–12. doi: 10.1038/s41375-023-01864-6 (PMC10169633; doi:10.1038/s41375-023-01864-6)
Supplement: Supplementary file 1 — Supplementary Material [file 41375_2023_1864_MOESM1_ESM.docx]

Supplemental Table 1. Patient and disease characteristics among patients with d and t-MDS and within subgroups of t-MDS according to type of primary disease *.

|  | **De novo-MDS**  **n=2283 (84%)** | **t-MDS**  **n=423 (16%)** | **Subgroups of t-MDS** | | |
| --- | --- | --- | --- | --- | --- |
|  |  |  | **Solid tumor n=176 (42%)** | **Hematological malignancy**  **n=160 (38%)** | **Non-malignant**  **n=63 (15%)** |
| **Median survival, months (95%CI)** | 31.1 (29.0-33.3) | 15.8 (13.6-18.1) | 22.3 (16.3-28.2) | 9.0 (7.1-10.9) | 26.1 (8.7-43.5) |
|  |  |  |  |  |  |
| **Sex** |  |  |  |  |  |
| Female | 922 (40%) | 194 (46%) | 98 (51%) | 62 (39%) | 27 (43%) |
| Male | 1361 (60%) | 229 (54%) | 87 (49%) | 98 (61%) | 36 (57%) |
|  |  |  |  |  |  |
| **Age at diagnosis, years** |  |  |  |  |  |
| <60 | 216 (9%) | 33 (8%) | 8 (5%) | 18 (11%) | 6 (10%) |
| 60-74 | 816 (36%) | 200 (47%) | 78 (44%) | 84 (53%) | 28 (44%) |
| ≥75 | 1251 (55%) | 190 (45%) | 90 (51%) | 58 (36%) | 29 (46%) |
|  |  |  |  |  |  |
| **Median age at diagnosis, years (range)** | 76 (16-97) | 73(18-92) | 75 (42-92) | 72 (18-91) | 72 (52-89) |
|  |  |  |  |  |  |
| **WHO subgroup** |  |  |  |  |  |
| MDS-SLD | 185 (8%) | 23 (5%) | 13 (7%) | 8 (5%) | 2 (3%) |
| MDS-MLD | 719 (32%) | 121 (29%) | 61 (35%) | 38 (24%) | 18 (29%) |
| MDS-RS** | 273 (12%) | 21 (5%) | 11 (6%) | 5 (3%) | 2 (3%) |
| MDS-EB-1 | 350 (15%) | 82 (19%) | 32 (18%) | 35 (22%) | 12 (19%) |
| MDS-EB-2 | 397 (17%) | 97 (23%) | 29 (17%) | 43 (27%) | 16 25%) |
| MDS with isolated del(5q) | 93 (4%) | 15 (4%) | 3 (2%) | 2 (1%) | 7 (11%) |
| MDS-U | 266 12%) | 64 (15%) | 27 (15%) | 29 (18%) | 6 (10%) |
|  |  |  |  |  |  |
| **Medullary blast count, %** |  |  |  |  |  |
| <2 | 730 (33%) | 108 (27%) | 50 (29%) | 31 (20%) | 21 (34%) |
| 2-4.9 | 668 (31%) | 110 (27%) | 54 (32%) | 38 (25%) | 14 (23%) |
| 5-9.9 | 384 (18%) | 91 (22%) | 35 (21%) | 42 (28%) | 11 (18%) |
| ≥10 | 407 (19%) | 98 (24%) | 32 (19%) | 42 (28%) | 15 (25%) |
| Missing | 94 | 16 | 5 | 7 | 2 |
|  |  |  |  |  |  |
| **IPSS-R cytogenetic risk group** |  |  |  |  |  |
| Very good | 165 (9%) | 25 (8%) | 9 (6%) | 4 (3%) | 8 (17%) |
| Good | 996 (56%) | 125 (38%) | 70 (50%) | 29 (24%) | 18 (38%) |
| Intermediate | 267 (15%) | 51 (16%) | 21 (15%) | 18 (15%) | 8 (17%) |
| Poor | 122 (7%) | 44 (13%) | 16 (11%) | 19 (16%) | 7 (15%) |
| Very poor | 216 (12%) | 84 (26%) | 25 (18%) | 51 (42%) | 7 (15%) |
| Missing | 517 | 94 | 35 | 39 | 15 |
|  |  |  |  |  |  |
| **IPSS-R** |  |  |  |  |  |
| Very low | 335 (19%) | 40 (12%) | 26 (18%) | 3 (3%) | 9 (19%) |
| Low | 564 (33%) | 72 (22%) | 36 (25%) | 19 (16%) | 12 (26%) |
| Intermediate | 337(19%) | 52 (16%) | 27 (19%) | 17 (15%) | 7 (15%) |
| High | 253(15%) | 77 (24%) | 27 (19%) | 37 (32%) | 9 (19%) |
| Very high | 250 (14%) | 83 (26%) | 26 (18%) | 41 (35%) | 10 (21%) |
| Missing | 544 | 99 | 34 | 44 | 17 |
|  |  |  |  |  |  |
| **Red blood cell transfusion dependency at diagnosis** |  |  |  |  |  |
| Yes | 1033 (45%) | 232 (55%) | 84 (48%) | 108 (68%) | 25 (40%) |
| No | 1243 (54%) | 190 (45%) | 92 (52%) | 51 (32%) | 38 (60%) |
| Missing | 7 | 1 | 0 | 1 | 0 |
|  |  |  |  |  |  |
| **Platelet transfusion dependency at diagnosis** |  |  |  |  |  |
| Yes | 125 (6%) | 47 (11%) | 13 (7%) | 27 (17%) | 3 (5%) |
| No | 2143 (94%) | 375 (89%) | 163 (93%) | 132 (83%) | 60 (95%) |
| Missing | 15 | 1 | 0 | 1 | 0 |
|  |  |  |  |  |  |
| **CCI** |  |  |  |  |  |
| 0 | 1361 (60%) | 222 (53%) | 90 (51%) | 98 (61%) | 18 (29%) |
| 1 | 506 (22%) | 108 (26%) | 51 (29%) | 34 (21%) | 18 (29%) |
| 2 | 230 (10%) | 51 (12%) | 19 (11%) | 19 (12%) | 11 (18%) |
| >2 | 186 (8%) | 42 (10%) | 16 (9%) | 9 (6%) | 16 (25%) |

*24 patients had an unknown primary disease

** Including both MDS-RS SLD and MDS-RS-MLD

Abbreviations: CI = Confidence interval, MDS-SLD = MDS with single lineage dysplasia, MDS-MLD = MDS with multilineage dysplasia, MDS-RS = MDS with ring sideroblasts, MDS-EB = MDS with excess blasts, MDS-U = MDS unclassifiable, CCI =Charlson Comorbidity Index, IPSS-R= International Prognostic Scoring System Revised

Supplemental table 2. Diseased t-MDS patients grouped according to their primary disease and the percentage of death where the primary disease was stated as the underlying cause of death.

| **Primary disease** | **Underlying cause of death the primary disease** |
| --- | --- |
| **Solid tumors** | 18/119 (15%) |
| Central nervous system/Eye | 3/7 (43%) |
| Head and neck | 2/5 (40%) |
| Lung | 3/14 (21%) |
| Prostate | 4/29 (14%) |
| Colon/rectal | 1/8 (13%) |
| Breast | 1/22 (5%) |
| Ovarian | 0/6 (0%) |
| Uterine | 0/13 (0%) |
|  |  |
| **Hematological malignancies** | 64/141 (45%) |
| Acute lymphocytic leukemia | 1/1 (100%) |
| Hodgkin lymphoma | 6/9 (67%) |
| Chronic lymphocytic leukemia | 6/9 (67%) |
| Myeloma | 12/19 (63%) |
| Non-Hodgkin lymphoma | 32/65 (49%) |
| Acute myeloid leukemia | 5/14 ((36%) |
| Myelofibrosis/MPN NOS | 1/5 (20%) |
| Polycythemia Vera | 1/8 (13%) |
| Essential thrombocythemia | 0/10 (0%) |

Abbreviations: MPN NOS = Myeloproliferative neoplasm not otherwise specified


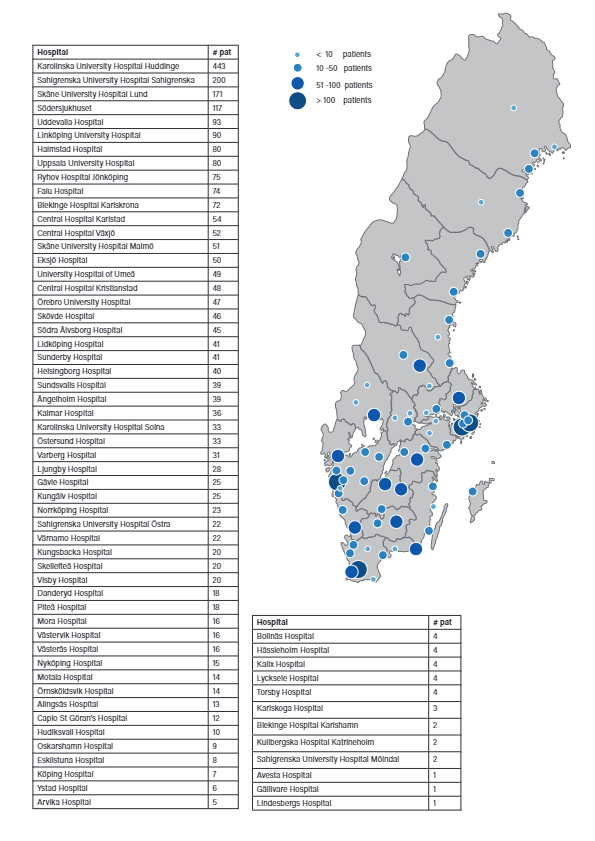
Supplemental Figure 1: Contributing sites, the number of patients from each hospital and their geographical distribution.


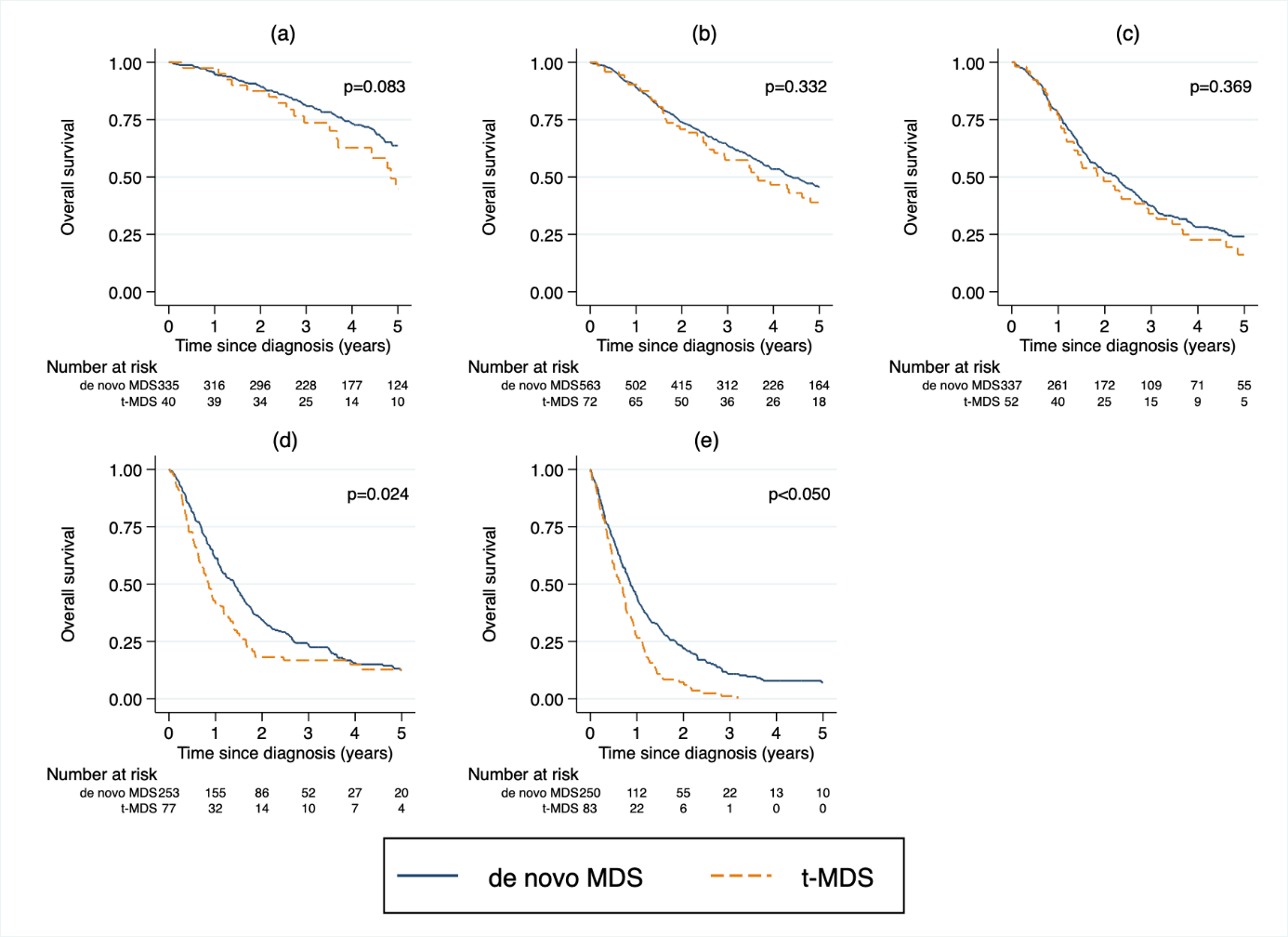


Supplemental Figure 2: OS in subgroups of each IPSS-R risk group in de novo and t-MDS

Figure 2a: OS of patients with IPSS-R very low by de novo or t-MDS

Figure 2b: OS of patients with IPSS-R low by de novo or t-MDS

Figure 2c: OS of patients with IPSS-R intermediate by de novo or t-MDS

Figure 2d: OS of patients with IPSS-R high by de novo or t-MDS

Figure 2e: OS of patients with IPSS-R very high by de novo or t-MDS


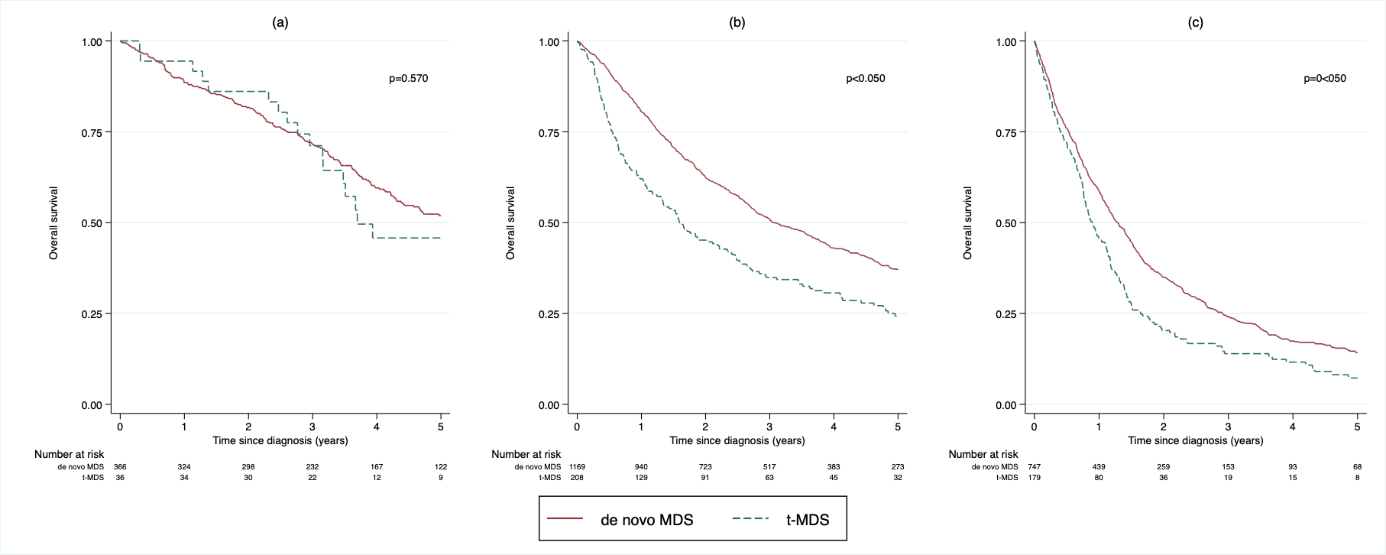


Supplemental Figure 3: OS in subgroups of each WHO group in de novo and t-MDS

Figure 3a: OS of patients with WHO good risk group by de novo or t-MDS

Figure 3b: OS of patients with WHO intermediate risk group by de novo or t-MDS

Figure 3c: OS of patients with WHO poor risk group by de novo or t-MDS

Appendix

For the purpose of the present study, we generated a dataset based on individual level record linkages between the Swedish MDS Register and several registers with national coverage at the National Board of Health and Welfare (Socialstyrelsen) including:

1. The Swedish National Patient Register (NPR). The NPR includes hospital discharge diagnoses according to International Classification of Diseases and covers all Swedish in-patient care from 1987 and onwards. Since 2001, diagnoses from specialized out-patient care are also recorded.^1^
2. The Swedish Cancer Register (SCR). The SCR was founded in 1958 and it is compulsory for all health care providers to report all newly detected malignant diseases to the register. The SCR contains detailed information coded according to the Systematized Nomenclature of Medical-Clinical Terms (SNOMED) and International Classification of Diseases (ICD). All registered cancers are classified according to ICD-7, as well as the ICD version (ICD-9 from 1987, and ICD-10 from 1993) that was used during the time of diagnosis. Since 2005 the International Classification of Diseases for Oncology (ICD-O/3) is used. The register is reported to be of high quality and has high completeness.^2^
3. The Swedish Prescribed Drug Register (PDR). The PDR was established in 2005 and includes all dispensed prescribed drugs in the Swedish population, no information on medications given at hospitals is available from this register.^3^ In the present study we used the PDR to find prescriptions of chemotherapy.
4. The Swedish Cause of Death register (CDR). The CDR is a virtually complete register of all deaths in Sweden since 1952.^4^ When a person dies the responsible physician is required to complete a mandatory cause of death certificate (including the main and contributing causes of death, as well as any other significant diseases). At the CDR one death cause is selected as the principal underlying cause of death, using an algorithm. This underlying cause of death was used in the present study.

Individual level record linkages were made possible by use of the unique personal identity number assigned to all residents in Sweden at birth or permanent residency. Information was also retrieved from the Population Register (PR) administered by Statistics Sweden (Statistiska Centralbyrån). The PR incudes all person registered in Sweden. Controls were randomly selected from the PR, matched 1:5 on age, sex and county of residence. Date of death or permanent emigration is automatically collected from the PR to the Swedish MDS Register and we used this information to calculate survival.

1. Ludvigsson JF, Andersson E, Ekbom A, et al: External review and validation of the Swedish national inpatient register. BMC Public Health 11:450, 2011

2. Barlow L, Westergren K, Holmberg L, et al: The completeness of the Swedish Cancer Register: a sample survey for year 1998. Acta Oncol 48:27-33, 2009

3. Wettermark B, Hammar N, Fored CM, et al: The new Swedish Prescribed Drug Register--opportunities for pharmacoepidemiological research and experience from the first six months. Pharmacoepidemiol Drug Saf 16:726-35, 2007

4. Brooke HL, Talback M, Hornblad J, et al: The Swedish cause of death register. Eur J Epidemiol 32:765-773, 2017
